# A Green Synthesis of CoFe_2_O_4_ Decorated ZIF-8 Composite for Electrochemical Oxygen Evolution

**DOI:** 10.3390/ijms24119585

**Published:** 2023-05-31

**Authors:** Atanu Panda, Hang-Kyu Cho, Hansang Kim

**Affiliations:** Department of Mechanical Engineering, Gachon University, Seongnam-si 13120, Gyeonggi-do, Republic of Korea; santu.atanu.panda@gmail.com (A.P.); 5318@gachon.ac.kr (H.-K.C.)

**Keywords:** spinel ferrite, ZIF-8, magnetic, CoFe_2_O_4_, oxygen evolution reaction

## Abstract

Low-cost, sustainable hydrogen production requires noble metal-free electrocatalysts for water splitting. In this study, we prepared zeolitic imidazolate frameworks (ZIF) decorated with CoFe_2_O_4_ spinel nanoparticles as active catalysts for oxygen evolution reaction (OER). The CoFe_2_O_4_ nanoparticles were synthesized by converting agricultural bio-waste (potato peel extract) into economically valuable electrode materials. The biogenic CoFe_2_O_4_ composite showed an overpotential of 370 mV at a current density of 10 mA cm^−2^ and a low Tafel slope of 283 mV dec^−1^, whereas the ZIF@CoFe_2_O_4_ composite prepared using an in situ hydrothermal method showed an overpotential of 105 mV at 10 mA cm^−2^ and a low Tafel slope of 43 mV dec^−1^ in a 1 M KOH medium. The results demonstrated an exciting prospect of high-performance noble metal-free electrocatalysts for low-cost, high-efficiency, and sustainable hydrogen production.

## 1. Introduction

Due to the increasing global demand for renewable energy and the effects of climate change, developing clean, environmentally sustainable energy sources has become extremely important in the twenty-first century [1,2,3]. Although sunlight and wind are abundant natural sources of renewable energy, their harvest requires highly sophisticated processing techniques and strongly depends on the geographical location [4,5]. Hydrogen is the primary sustainable source of renewable energy. Since the last century, water electrolysis has emerged as a promising method of producing high-purity hydrogen. Due to the increasing availability of electricity generated from renewable sources, water electrolysis has recently gained immense attention in hydrogen production.

The general water-splitting reaction comprises two half-responses: i.e., the oxygen evolution reaction (OER) at the anode and the hydrogen evolution reaction (HER) at the cathode [6,7].

HER       2H^+^ + 2e^−^ = H_2_ (1)

    OER    2H_2_O = O_2_ + 4H^+^ + 4e^−^

   Overall   2H_2_O = O_2_ + 2H_2_

Both the HER and OER are kinetically favorable and require some overpotentials to achieve the desired reaction rate. Water splitting theoretically requires a Gibbs free energy (G) of approximately 237.2 kJ mol^−1^ to enable the thermodynamically uphill reaction in the electrolyzer [8]. However, the unfavorable thermodynamics and the resulting large overpotential of water electrolysis limit large-scale hydrogen generation [9,10]. The highest water splitting efficiency to date was reported using noble metal-based electrocatalysts, particularly Pt-based catalysts, for the HER and Ir/Ru-based catalysts for the OER [11,12,13]. Nonetheless, the scarcity and high cost of precious metals significantly restrict their applications in commercial water splitting. Therefore, the development of non-precious alternatives with excellent activity and durability is imperative [14].

Biogenic materials are drawing increasing attention due to their sustainability. Some biogenic materials are renewable and can be obtained from waste products, which significantly reduces their production cost [15,16,17]. More importantly, biogenic materials can be engineered to have specific catalytic properties, making them useful for selective reactions [15]. These materials become environmentally friendly alternatives to synthetic catalysts because they are biodegradable and non-toxic. In addition, biogenic materials exhibit improved stability and activity compared to synthetic catalysts, suitable for specific applications in fuel cells and water splitting. Overall, using biogenic materials in electrocatalysis can potentially contribute to the development of more sustainable and environmentally friendly technologies.

Metal–organic frameworks (MOFs) [18,19], which are composed of organic ligands and metal ions or clusters, are a new class of highly porous materials with high crystallinity and a long-range order [20]. Numerous MOFs have been employed for electrochemical water splitting due to their intrinsic properties, such as their large surface area, customizable chemical components, configurable pore architectures, and various topologies [21,22,23,24]. Additionally, the characteristics of MOFs can be modified by coupling them with a variety of functional materials, including polyoxometalates, metal compounds, carbon nanotubes (CNTs), and other conductive substrates to generate MOFs or MOF/substrate composites [25,26,27,28,29,30]. The excellent water-splitting performance of such composites can be attributed to their increased active sites and conductivity due to functionalization. Furthermore, the MOF-based template enables the molecular and atomic-level rearrangement of the components during stacking with other materials. Over the past decades, various MOF-based electrocatalysts have been reported. Among them, zeolite imidazolate framework (ZIF), composed of imidazole linkers coordinated to transition metals, such as Zn^2+^, is a suitable MOF to support heterogeneous catalysts because its large pore size can allow the transport of large reactant and product molecules [31,32]. Moreover, the metal centers within the ZIF-8 structure can undergo redox reactions, enabling efficient electron transfer during electrochemical reactions. This redox activity contributes to the catalytic performance of ZIF-8 as an electrocatalyst, facilitating the desired electrochemical transformations. The unique structural features of ZIF-8, such as the presence of confined nanocages and open metal sites, allow for selective catalytic activity towards target reactions. This selectivity, combined with the high catalytic efficiency, makes ZIF-8 electrocatalysts promising for energy conversion and storage applications [33,34,35]. The porous nature of ZIF-8 facilitates the efficient mass transport of reactants and products. Its interconnected framework enables the diffusion of species through the catalyst, improving reaction kinetics and minimizing concentration polarization [33,36]. On the other hand, magnetic materials are ideal catalyst supports as they can be recovered easily without centrifugation or filtration [37]. However, pure magnetic nanoparticles tend to aggregate and cannot disperse uniformly throughout the reaction system. Incorporating magnetic nanoparticles into porous materials can prevent their aggregation and create a magnetic heterogeneous catalyst [32,37,38,39]. The magnetic composites have several advantages, including catalytically active surfaces and a magnetic separation capability. Moreover, some magnetic nanoparticles have multiple oxidation states and are expected to be electrocatalytically active, such as NiCo_2_O_4_ [40], NiMoO_4_ [41], Co_3_O_4_ [42], NiO [43], and MnO_2_ [44]. Spinel ferrites, denoted as MFe_2_O_4_ (M = Mn, Co, Ni, Zn, Cu, etc.), are a type of binary/ternary metal oxide. These ferrites are highly desirable because of their low cost, earth abundance, magnetism, catalytic activity, and conductivity [45,46,47]. They are composed of Fe, Co, and Ni, which are typically used to fabricate highly efficient OER catalysts [48,49,50,51]. However, only a few studies have focused on the application of spinel ferrites as OER electrocatalysts. For instance, Guo et al. prepared a series of MFe_2_O_4_ (M = Co, Ni, Cu, and Mn) spinel nanofibers for the OER. Among them, CoFe_2_O_4_ showed the lowest overpotential of 408 mV at the current density of 5 mA cm^−2^ [46]. Furthermore, polyaniline CNTs and carbon were used to modify the spinel CoFe_2_O_4_, and their overpotentials decreased to 314 and 378 mV, respectively [52,53]. Additionally, in 2017, Lu et al. reported a MOF-based CoFe_2_O_4_/C composite with an overpotential of 240 mV, which is one of the lowest values reported for ferrite-based catalysts [54]. However, this overpotential is still too high for a practical OER.

**Scheme 1:**
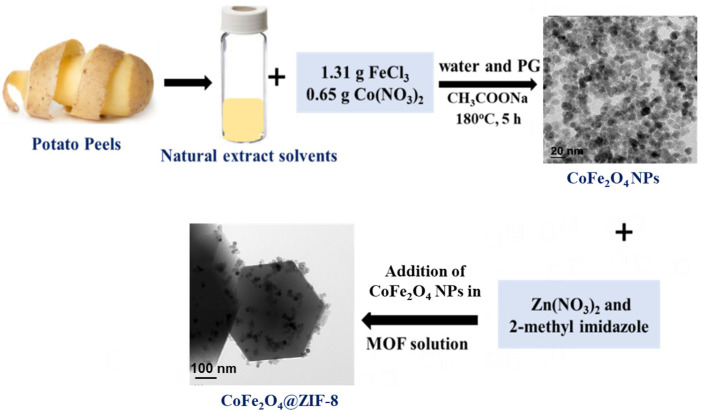
Scheme for synthesis of CoFe_2_O_4_@ZIF-8.

Inspired by these observations, herein, we report a unique strategy to utilize food waste to synthesize biogenic electrode materials in a cost-effective, eco-friendly, and simple manner (Scheme 1). Potato peels were used as a precursor for the synthesis of ZIF@CoFe_2_O_4_ composites. According to the US import/export report, South Korea produces approximately 530,000 metric tons of potatoes annually. Potato peel comprises starch, polysaccharide, protein, acid-soluble and acid-insoluble lignin, and lipids. The lipid fraction contains long-chain fatty acids, alcohols, triglycerides, and sterol esters. This composition can be used as a natural polymeric precursor. Therefore, waste potato peels can be a great source of chemical precursor, such as biogenic surfactant, for the synthesis of metal nanoparticles that can control the growth of the NPs and inhibit aggregation. Therefore, waste potato peels can be a great source of biogenic precursors for the synthesis of metal nanoparticles. Interestingly, the synthesized ZIF@CoFe_2_O_4_ composite showed excellent electrocatalysis toward the OER in a 1 M KOH solution. The enhanced catalytic activity of the composite was attributed to the synergistic interaction between the transition metals Co–Fe and Zn. This facile approach may open up new opportunities for the utilization of bio-waste in the preparation of well-defined nanostructures.

## 2. Results and Discussion

### 2.1. Characterization

**Figure 1:**
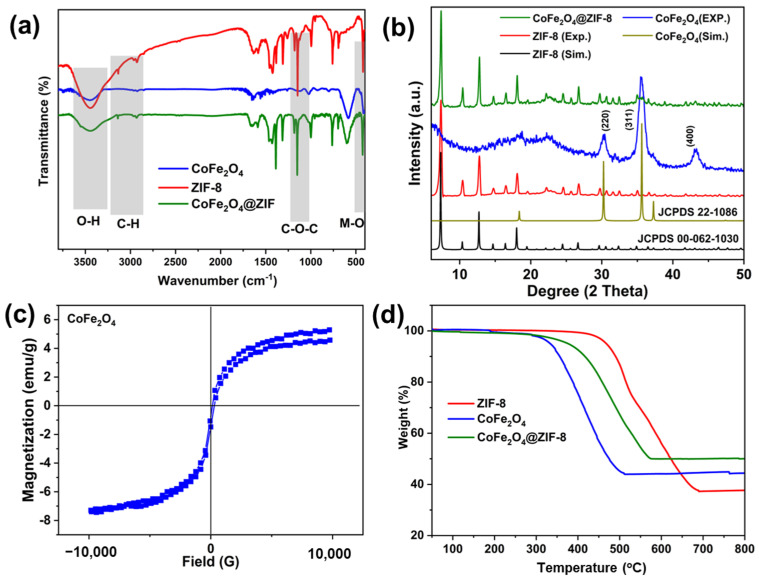
(**a**) FT-IR spectra; (**b**) powder XRD patterns; (**c**) VSM analysis; (**d**) TGA curves of the CoFe_2_O_4_, ZIF-8, and CoFe_2_O_4_@ZIF-8 samples.

Figure 1a displays the Fourier-transform infrared (FT-IR) spectra of the ZIF-8, CoFe_2_O_4_, and CoFe_2_O_4_@ZIF-8 samples. All the samples showed a broad band at approximately 3500 cm^−1^ corresponding to the hydroxyl group [37,53,55]. The band at approximately 3000 cm^−1^ corresponded to the stretching of the C–H bond. The peak at approximately 1150 cm^−1^ indicated the presence of the carboxylic/C–O–C group [37,55]. The stretching vibration of the imidazole ring in ZIF-67 was observed at 605–1450 cm^−1^. Further, the stretching vibration near 3400 cm^−1^ was related to N–H and –OH groups. The CoFe_2_O_4_ and CoFe_2_O_4_@ZIF-8 samples showed metal oxide peaks at approximately 520 cm^−1^, indicating the successful incorporation of CoFe_2_O_4_ into ZIF-8 [54]. Furthermore, powder X-ray diffraction (XRD) was conducted to obtain the diffraction patterns of the ZIF-8, CoFe_2_O_4_, and CoFe_2_O_4_@ZIF-8 samples (Figure 1b). The diffraction pattern of ZIF showed a series of strong diffraction peaks at 2θ = 7.30, 10.35, 12.70, 14.80, and 16.40°, corresponding to the (110), (200), (211), (220), (310), and (222) planes of Zn, respectively [37]. The diffraction peaks for the pure spinel cobalt ferrite phase could be indexed to those in PDF #4-006-4147 [56]. In addition, several peaks corresponding to α-Fe_2_O_3_ and Co_3_O_4_ were observed in Figure 1b, implying that α-Fe_2_O_3_ and Co_3_O_4_ impurities were formed during the synthesis of CoFe_2_O_4_ [53,54]. For example, the weak diffraction peaks at 2θ = 31.27 and 36.84° were associated with the (220) and (311) planes of cubic Co_3_O_4_ (PDF #04-005-4386) [54].

Figure S1 shows the adsorption-desorption isotherms of the CoFe_2_O_4_, ZIF-8, and the CoFe_2_O_4_/ZIF-8 composite. The CoFe_2_O_4_/ZIF-8 had a larger Brunauer–Emmett–Teller (BET) area than CoFe_2_O_4_ due to the large surface area of ZIF-8. The specific BET surface areas of the CoFe_2_O_4_, ZIF-8, and CoFe_2_O_4_/ZIF-8 were approximately 620, 1040, and 580 m^2^ g^−1^, respectively. ZIF-8 showed a typical type-I nitrogen adsorption–desorption isotherm [32]. The list of average pore diameter from BET measurements have been summarized in Table S2.

The thermogravimetric analysis (TGA) of the CoFe_2_O_4_, ZIF-8, and CoFe_2_O_4_/ZIF-8 composite was performed in a nitrogen atmosphere. As shown in Figure 1d, the TGA curve of ZIF-8 exhibited a total weight loss of 75% up to 700 °C. In contrast, the weight loss of CoFe_2_O_4_ was only 40%, which was attributed to the elimination of the H_2_O adsorbed on the particle surfaces. Moreover, in the initial stage, the weight loss of CoFe_2_O_4_/ZIF-8 (31%) was caused by the vaporization of the adsorbed water or methanol. Due to the rapid disintegration of the ZIF-8 molecules, significant weight loss was observed at 250–500 °C. When the temperature reached 600 °C, the ZIF-8 molecules transformed completely to ZnO.

Figure 1c illustrates the room-temperature magnetic behavior of CoFe_2_O_4_ and its composite measured using a vibrating sample magnetometer (VSM) in the −10,000–10,000 G magnetic field range. Bare CoFe_2_O_4_ exhibited ferromagnetic behavior with nonzero remnant magnetization (Mr) and coercive force (Hc), and its saturation magnetization was 6.36 emu g^−1^. In contrast, the saturation magnetization of CoFe_2_O_4_@ZIF-8 (Figure S2) was 4.009 emu g^−1^. The decreased magnetization value in the composite was because of the non-magnetic nature of ZIF-8.

The optical reflectance spectra were measured using an ultraviolet diffuse reflectance spectroscopy (UV-DRS, JASCO, Kingsgrove, NSW, Australia, V-770) with an integrating sphere attachment. CoFe_2_O_4_ and CoFe_2_O_4_@ZIF-8 were mixed with tetrahydrofuran (THF) and subjected to ultrasonic dispersion. The UV–Vis absorption spectra were recorded from 200 to 800 nm (Figure S4). The spectra of CoFe_2_O_4_ and CoFe_2_O_4_@ZIF-8 were similar, with peaks at 312 nm and 349 nm, respectively. Also, as a reference, absorbance was analyzed using FT-IR (Figure S5) and peak position is matching with the Figure 1a Ft-IR in reflectance mode. 

**Figure 2:**
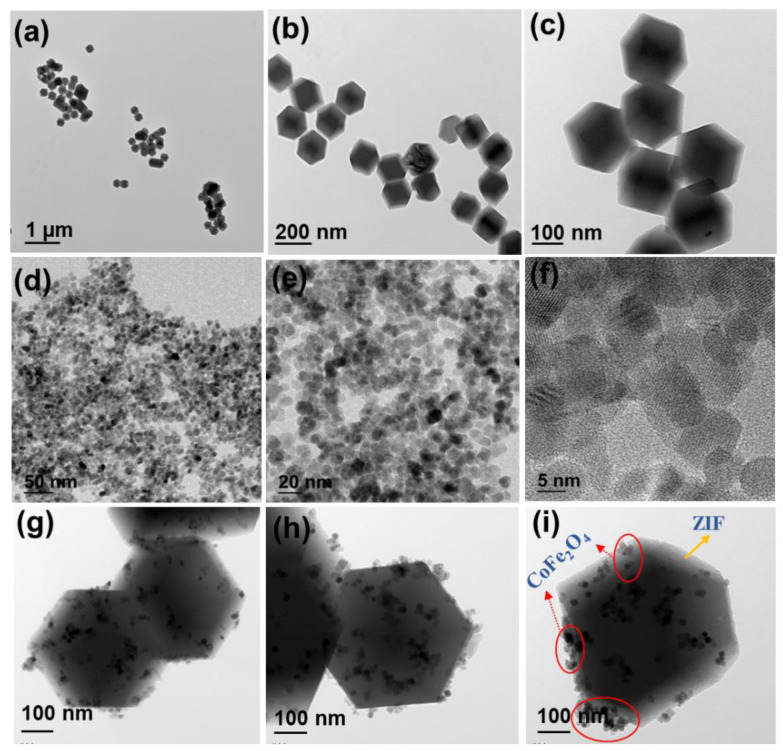
TEM, HRTEM images of (**a**–**c**) ZIF-8, (**d**–**f**) CoFe_2_O_4_, and (**g**–**i**) CoFe_2_O_4_@ZIF-8.

The morphology analysis was performed using transmission electron microscopy (TEM). Figure 2a–c shows the TEM images of the ZIF-8 synthesized in this work, wherein a homogeneous and hierarchical structure was observed with regular polyhedron shapes. Additionally, the as-synthesized ZIF-8 exhibited no obvious aggregation, and its particle size varied between 100 and 140 nm. The hierarchical porous structures are favorable for improving the ionic conductivity of the composite materials.

**Figure 3:**
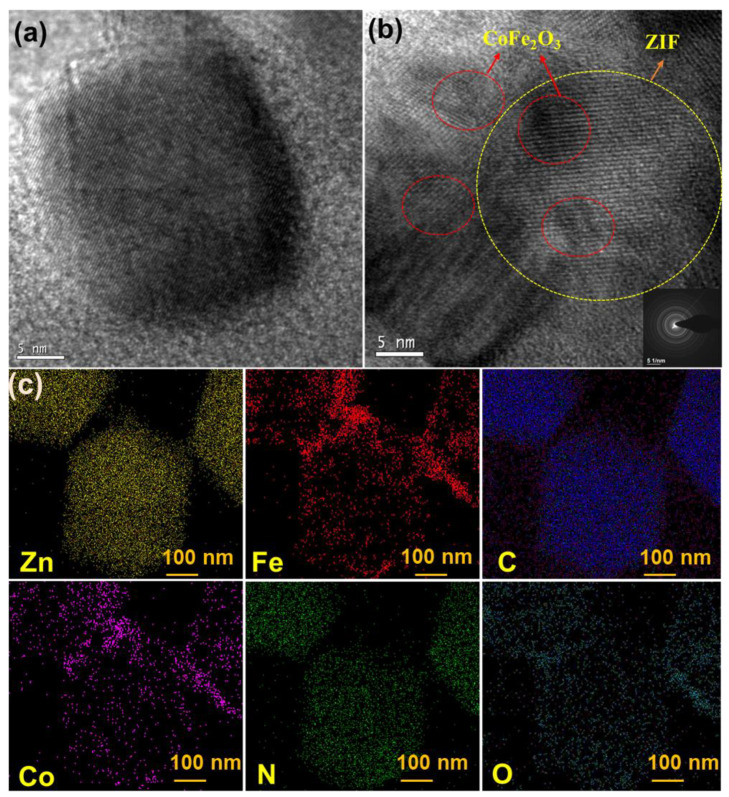
HR-TEM images of (**a**) CoFe_2_O_4_ and (**b**) CoFe_2_O_4_@ZIF-8, (Red circles are CoFe_2_O_4_ and yellow circle is ZIF) and (**c**) Elemental mapping of CoFe_2_O_4_@ZIF-8.

The TEM and high-resolution TEM (HR TEM) images in Figure 2d–f and Figure 3a illustrate the CoFe_2_O_4_ heterodimer nanoparticles with a spherical Co component (8–9 nm, darker contrast) attached to a rhombohedral Fe_2_O_3_ domain (13–15 nm, brighter contrast). Figure 2g–i and Figure 3b show the TEM and HR TEM images of the CoFe_2_O_4_@ZIF-8 composite. The CoFe_2_O_4_ nanoparticles grew on the surface of the ZIF framework. However, a slight agglomeration of the CoFe_2_O_4_ nanoparticles was observed on the ZIF surface, likely due to their magnetic property [37]. The energy-dispersive X-ray spectroscopy (EDS) analysis and mapping in Figure 3c showed the uniform distribution of the main elements Zn, C, N, and O throughout the MOF, whereas the elemental Fe and Co were only detected on the surface. The corresponding scanning electron microscopy (SEM)–EDS of the controlled samples is provided in Figure S3 and Table S1.

### 2.2. Oxygen Evolution Reaction (OER)

**Figure 4:**
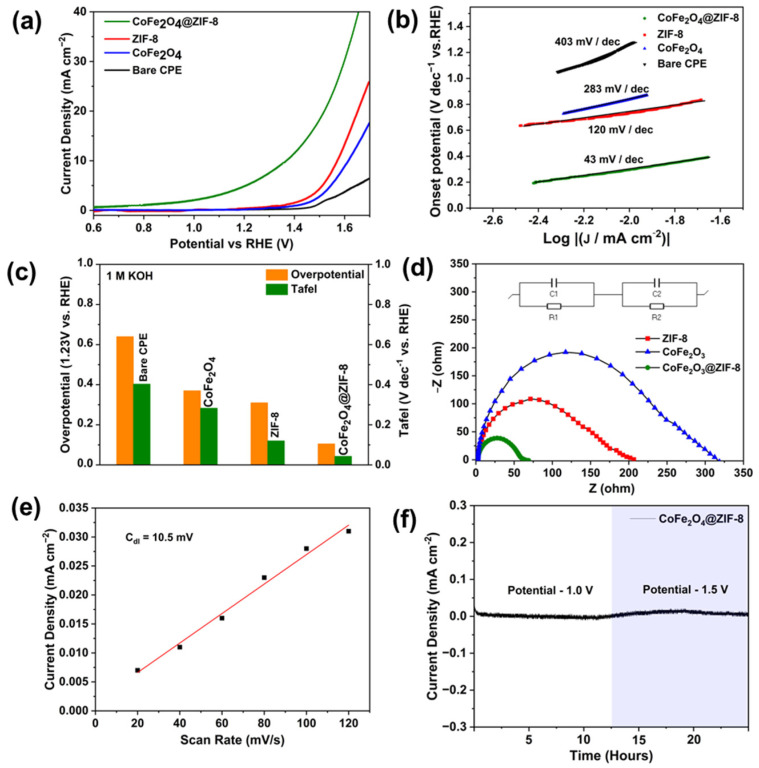
(**a**) OER polarization curves; (**b**) Tafel plots, with 75% *iR* correction; (**c**) Comparative overpotentials at 0.2 and 10 mA/cm^2^; (**d**) Nyquist plots, (**e**) Linear plot of CV at different scan rates (20–120 mV) (**f**) chronoamperometry stability of CoFe_2_O_4_@ZIF-8 at potentials of 1 V and 1.5 V in 1 M KOH.

In this study, we used a three-electrode system to perform water oxidation, and *iR* correction was applied to the Tafel plots and polarization curves. To assess the bifunctional electrocatalytic activity of the synthesized catalysts, linear sweep voltammetry (LSV) measurements were performed for the OER in a basic electrolyte (1 M KOH) over the potential range of 0.00–1.5 vs. a reversible hydrogen electrode (RHE) (Figure 4a). As a reference, a catalytic current density of −10 mA cm^−2^ was used because this is the typical value for photoelectrical cells with a solar capacity of ~10% [14,15,16].

The blank carbon paper electrode (CPE) was not active for OER, whereas the ZIF-8 electrode showed a current of 20 mA at 1.5 V vs. RHE. In contrast, the cathodic current for CoFe_2_O_4_@ZIF-8 shifted toward a more positive potential and reached 40 mA at 1.5 V vs. RHE. However, the LSV curve of the pure ZIF-8 and CoFe_2_O_4_ had some fluctuating signals due to their overly compensated *iR*. Moreover, Figure 4c shows that CoFe_2_O_4_@ZIF-8 required a much lower overpotential (105 mV) than CoFe_2_O_4_ (370 mV) and ZIF-8 (310 mV) to achieve a current density of 10 mA cm^−2^. The lowest overpotential for CoFe_2_O_4_@ZIF-8 composite was attributed to the synergistic interaction between Zn and the transition metals present in CoFe_2_O_4_. CoFe_2_O_4_ decoration increased the charge transfer capability of ZIF-8, decreased the energy barrier of the CoFe_2_O_4_@ZIF-8 composite, and accelerated the OER kinetics. CoFe_2_O_4_@ZIF-8 exhibited the lowest onset potential of 1.35 V among all the catalysts investigated (1.59 V for ZIF-8). This indicated that the oxygen ion adsorption capacity of CoFe_2_O_4_@ZIF-8 increased, and the catalytic activity toward the OER was improved because of the synergistic effect between the transition metals. Table 1 summarizes the overpotentials and Tafel slope values of the electrode materials. The combination of CoFe_2_O_4_ nanoparticles with ZIF-8 in a composite structure can lead to synergistic effects. CoFe_2_O_4_ nanoparticles possess catalytic activity for OER, while ZIF-8 can provide a high surface area and facilitate reactant diffusion. The composite structure allows for the integration of both materials’ properties, resulting in improved electrocatalytic performance.

As shown in Figure 4b, CoFe_2_O_4_@ZIF-8 possessed the lowest Tafel slope (43 mV/dec), possibly because of the strong electron coupling between Co, Fe, and Zn. In contrast, the Tafel slopes of the ZIF-8 and CoFe_2_O_4_ were 120 and 283 mV/dec, respectively. The lowest Tafel slope of CoFe_2_O_4_@ZIF-8 indicated that it had the fastest charge transfer kinetics during the OER, and the rate-determining step followed the Volmer OER mechanism. The OER activity of the CoFe_2_O_4_@ZIF-8 catalyst was compared with those of previously reported Co–Fe metal oxide-based catalysts (Table 2). The CoFe_2_O_4_@ZIF-8 composite is a promising high-performance OER electrocatalyst because of its low overpotential and Tafel slope.

As shown in the Nyquist plots (Figure 4d) obtained through an electrochemical impedance spectroscopy (EIS), the CoFe_2_O_4_@ZIF-8 composite exhibited a smaller semicircle than the other two catalysts, indicating its higher conductivity, which could positively affect the electrocatalytic activity of an electrode. The Nyquist plots of the catalysts were fitted using EC laboratory software (Version B11.41). An equivalent circuit consisting of an Ohmic resistance (*R_s_*), charge transfer resistance (*R_ct_*), and constant phase element (Q) was built. The space charge layer capacitance (C) of the composite was calculated using the Brugg Equation (2).

C = (*R*·Q)^1/α^/*R*

(2) $$\frac{1}{R}=\frac{1}{R_{S}}+\frac{1}{R_{ct}}$$

The *R_ct_* value of ZIF-8 (210 Ω) was lower than that of CoFe_2_O_4_@ZIF-8 (57 Ω). The CoFe_2_O_4_@ZIF-8 composite showed the lowest impedance, indicating that the synergistic effect of Co and Fe effectively improved the charge transport kinetics of the MOF.

The electrochemical active surface area (ECSA) plays an important role in electrocatalysis. To calculate the ECSA, cyclic voltammetry (CV) was measured with different scan rates in a non-faradic potential window of 0–0.25 V. The double layer capacitance (C_dl_) of the bare electrode, ZIF-8, CoFe_2_O_4_, and CoFe_2_O_4_@ZIF-8 composite was calculated by plotting the current density between anodic and cathodic sweep vs. the scan rate (20–120 mV).

As shown in Figure 4e and Figure S6, the fitted slope is twice the C_dl,_ and all the fitted graphs show high linearity. The ECSA was calculated by using the bellow equation: ECSA = C_dl_/C_s_, where C_s_ is the specific capacitance in an alkaline electrolyte (0.040 mF·cm^−2^). CoFe_2_O_4_@ZIF-8 composite had a higher ECSA (260) than bare electrode (0.95), ZIF-8 (85), and CoFe_2_O_4_ (35), indicating a higher density of catalytic active sites in CoFe_2_O_4_@ZIF-8 due to the synergistic interaction between Zn, Fe, and Co.

The long-term stability of the CoFe_2_O_4_@ZIF-8 composite electrode was tested using a chronoamperometry method at two different potentials (1 V and 1.5 V). At 1 V, the electrode material was stable for up to 10 h. After increasing the potential to 1.5 V, a significant increment in current was observed (Figure 4f). Post-OER stability characterization showed that the crystallinity and morphology of the CoFe_2_O_4_@ZIF-8 composite electrode remained unchanged (Figure S7).

## 3. Materials and Methods

### 3.1. Materials

All organic solvents were purchased from local companies (Daejung Chemicals, and Samchun Chemicals, Gyeonggi-Do, Republic of Korea). The other chemicals were purchased from Sigma Aldrich. All the chemicals used were of an analytical grade and used without further purification. All the experiments were performed using deionized (DI) water.

### 3.2. Instrumentation

Wide-angle powder XRD analysis was performed on a Rigaku diffractometer (X-ray generator output: 3 kW, target material: Cu, 20–60 kW, 2–60 mA) at a scan rate of 4 °min^−1^. Field-emission SEM (Jeol, Tokyo, Japan, JSM-7500F, 1.0 nm at 15 kV) and TEM (Tecnai 2010, Oregon, United States, at a voltage of 300 kV and a resolution of 1.4) were used to examine the morphologies of the catalysts. HR resolution images were achieved using a fully embedded high-angle annular dark field detector. All the electrochemical experiments were conducted on a ZIVE SP1 compact electrochemical workstation equipped with smart manager software. The N_2_ adsorption isotherm measurements were performed using a Micromeritics ASAP-2020 system to calculate their specific surface areas and pore diameters. High-purity N_2_ gas (99.999%) was used for the measurements. TGA was performed using a Scinco TGA N-1000 instrument at a heating rate of 20 °C min^−1^.

### 3.3. Synthesis of ZIF-8

Typically, 0.8 g of Zn (NO_3_)_2_∙H_2_O was dissolved in 40 mL of methanol in a 50 mL beaker. Then, 0.526 g of 2-methyl imidazole was dissolved in 40 mL methanol. These two solutions were mixed and transferred to a 100 mL autoclave, followed by stirring at room temperature [37]. The resultant suspension was then transferred to a preheated oven at 100 °C for 12 h. Finally, the mixture was collected from the autoclave and washed with methanol and water repeatedly through centrifugation at 10,000 rpm and dried at 80 °C to obtain the ZIF-8 power.

### 3.4. Extraction of Potato Peels and the Synthesis of Spinel CoFe_2_O_4_

The collected potato peels were washed with DI water to remove any impurities and dried in a hot air oven at 40 °C. The dried potato peels were cut into fine pieces and crushed in a mortar to a powder form. The powdered peels (20 g) were mixed with 200 mL of water in a round bottle flask. The flask was refluxed at 80 °C for 12 h to obtain a pale yellowish slurry (Scheme 1). Later, this yellowish slurry was centrifuged and filtered through a cheesecloth. The resulting filtrate was used as a natural biogenic precursor for the synthesis of CoFe_2_O_4_ nanoparticles. To synthesize the spinel CoFe_2_O_4_, 1.31 g of FeCl_3_ and 0.65 gm of Co (NO_3_)_2_ were dissolved in a mixture of 10 mL of DI water and 30 mL of the biogenic precursor. The resulting mixture was stirred for 30 min at room temperature to obtain a homogeneous solution. The reducing agent, CH_3_COONa (2.45 g), was then added to the homogeneous solution under stirring. The resulting solution was transferred to a 50 mL autoclave and kept in a preheated oven at 180 °C for 10 h. Then, the obtained product was extracted and washed with ethanol several times to remove unreacted chemicals. Subsequently, the resulting particles were separated using a magnetic bar. The final product was obtained after drying at 100 °C overnight.

### 3.5. Synthesis of CoFe_2_O_4_@ZIF-8

To synthesize the CoFe_2_O_4_@ZIF-8 composite, 0.65 g of FeCl_3_ and 0.32 g of Co(NO_3_)_2_ were dissolved in 30 mL of the biogenic potato peel precursor, followed by adding 1.2 g of CH_3_COONa. Under stirring at room temperature, 0.7 g of the pre-synthesized ZIF-8 was added gradually, and the mixture was stirred for another 30 min. (Scheme 1) Finally, the resulting mixture was transferred to a 50-mL steel autoclave and heated at 180 °C for 10 h. The resulting product was decanted through centrifugation, followed by washing with ethanol and water. The final product was separated from the solution using a magnet bar and dried under vacuum at 100 °C for 12 h.

### 3.6. Electrochemical Experiments

All the electrochemical measurements were performed in a three-electrode system in 1 M KOH. A CPE was used as the working electrode, whereas Pt and Ag/AgCl were used as the counter and reference electrodes, respectively. To prepare the working electrode, 7 mg of the synthesized catalyst was dispersed in a mixture of 500 µL of ethanol, 485 µL of water, and 15 µL of Nafion, where ethanol and Nafion were used as the drying agent and binder, respectively. The mixed solution was ultrasonicated to obtain a homogeneous ink. Subsequently, 30 µL of the homogeneous ink was drop-cast and loaded onto a 1 cm^2^ area of a previously cleaned CPE. Drop-casting was performed to modify the CPE with a catalyst loading of 0.2 mg cm^−1^. The modified electrodes were dried for 10 h in a hot air oven at 80 °C. All the polarization experiments were conducted at a scan rate of 5 mV s^−1^ over a potential window of 0–1.5 V. EIS measurements were performed in the frequency range from 100 kHz to 0.1 Hz. The capacitances of the double layers were determined using a cyclic voltmeter over the potential window of 0.0–0.2 V, and the scan rate was gradually increased from 20 to 120 mV s^−1^.

## 4. Conclusions

Inspired by nature, we developed a unique, cost-effective method to synthesize biogenic CoFe_2_O_4_-decorated ZIF-8 composites for highly effective OER. A potato-peel-derived extract was used as a natural precursor for the synthesis of the CoFe_2_O_4_ particles. The average particle size of CoFe_2_O_4_ was approximately 15 nm. The spherical CoFe_2_O_4_ particles and highly porous ZIF-8 in the CoFe_2_O_4_@ZIF-8 created many catalytically active sites and facilitated the diffusion of the electrolyte ions to the active electrode. The synthesized CoFe_2_O_4_@ZIF-8 composite exhibited a low onset potential (0.5 V at the current density of 10 mA cm^−1^). In addition, the composite demonstrated excellent cycle stability and chronoamperometric stability for up to 10 h and retained 95% of its initial capacity. The overpotential of the CoFe_2_O_4_@ZIF-8 composite was 105 mV, the lowest value reported for spinel metal oxide materials. Thus, the eco-friendly synthesis approach proposed in this study paves the way for preparing magnetic materials from biogenic waste. Overall, the current research on CoFe_2_O_4_@ZIF-8 as an electrocatalyst highlights its promising prospects in energy conversion and storage technologies. Its unique composite structure and synergistic effects provide new opportunities for designing efficient and stable electrocatalysts, contributing to the development of sustainable energy solutions. Furthermore, because the CoFe_2_O_4_@ZIF-8 composite prepared in this study shows high electrocatalytic efficiency and low overpotential, the synthesis method is a potential approach for preparing green renewable energy sources.

## Figures and Tables

**Table 1:** Comparison of ECSA, overpotential, and Tafel slopes for synthesized catalysts during OER in 1 M KOH.

Catalyst	OER		
	ECSA (cm^2^)	Overpotential at −10 mA cm^−2^ (mV)	Tafel (mV dec^−1^)
**CPE**	0.95	151.1	403
**CoFe_2_O_4_**	35	370	283
**ZIF-8**	85	310	120
**CoFe_2_O_4_@ZIF-8**	260	105	43

**Table 2:** Comparison of the OER performance of various catalysts according to the Tafel slope (mV/dec) and overpotential (mV).

Catalyst	b (mVdec^−1^)	Overpotential at 10 mA cm^−2^ (η10 (mV))	Reference
CoFe_2_O_4_ (powders)	45	295	[57]
CoFe_2_O_4_ (powders)	126	435	[58]
CoFe_2_O_4_ (hollow nanofibers)	95	414	[59]
CoFe_2_O_4_ (thin films)	54	490	[60]
CoFe_2_O_4_ (nanoparticles)	73	387	[53]
CoFe_2_O_4_ (nanoplates)	61	360	[61]
CoFe_2_O_4_ (nanofibers)	107	340	[62]
Co_3_S_4_/EC–MOF	120	226	[63]
S–CoFe–PBA/CFP	35.2	235	[64]
FeMOFs-SO_3_	36.2	218	[65]
CoFe–MOF–OH		310	[66]
Fe (OH)_3_@CoMOF-74		292	[67]
Fe–Co–P Hollow Sphere	33	252	[68]
**ZIF@CoFe_2_O_4_**	**43**	**105**	**This work**

## Data Availability

The data that support the findings of this study are openly available at the request of the reader.

## Supplementary Materials

The following supporting information can be downloaded at: https://www.mdpi.com/article/10.3390/ijms24119585/s1.

## References

[1] Kanan M.W., Surendranath Y., Nocera D.G. (2009). Cobalt–Phosphate Oxygen-Evolving Compound. Chem. Soc. Rev..

[2] Lewis N.S., Nocera D.G. (2006). Powering the Planet: Chemical Challenges in Solar Energy Utilization. Proc. Natl. Acad. Sci. USA.

[3] Cook T.R., Dogutan D.K., Reece S.Y., Surendranath Y., Teets T.S., Nocera D.G. (2010). Solar Energy Supply and Storage for the Legacy and Nonlegacy Worlds. Chem. Rev..

[4] Xiang C., Papadantonakis K.M., Lewis N.S. (2016). Principles and Implementations of Electrolysis Systems for Water Splitting. Mater. Horiz..

[5] Coridan R.H., Nielander A.C., Francis S.A., McDowell M.T., Dix V., Chatman S.M., Lewis N.S. (2015). Methods for Comparing the Performance of Energy-Conversion Systems for Use in Solar Fuels and Solar Electricity Generation. Energy Environ. Sci..

[6] Suen N.T., Hung S.F., Quan Q., Zhang N., Xu Y.J., Chen H.M. (2017). Electrocatalysis for the Oxygen Evolution Reaction: Recent Development and Future Perspectives. Chem. Soc. Rev..

[7] Anantharaj S., Ede S.R., Sakthikumar K., Karthick K., Mishra S., Kundu S. (2016). Recent Trends and Perspectives in Electrochemical Water Splitting with an Emphasis on Sulfide, Selenide, and Phosphide Catalysts of Fe, Co, and Ni: A Review. ACS Catal..

[8] Jiang Y., Lu Y. (2020). Designing Transition-Metal-Boride-Based Electrocatalysts for Applications in Electrochemical Water Splitting. Nanoscale.

[9] Li X., Hao X., Abudula A., Guan G. (2016). Nanostructured Catalysts for Electrochemical Water Splitting: Current State and Prospects. J. Mater. Chem. A Mater..

[10] Fang M., Dong G., Wei R., Ho J.C. (2017). Hierarchical Nanostructures: Design for Sustainable Water Splitting. Adv. Energy Mater..

[11] Suryanto B.H.R., Wang Y., Hocking R.K., Adamson W., Zhao C. (2019). Overall Electrochemical Splitting of Water at the Heterogeneous Interface of Nickel and Iron Oxide. Nat. Commun..

[12] Hu E., Feng Y., Nai J., Zhao D., Hu Y., Lou X.W. (2018). Construction of Hierarchical Ni-Co-P Hollow Nanobricks with Oriented Nanosheets for Efficient Overall Water Splitting. Energy Environ. Sci..

[13] You B., Sun Y. (2018). Innovative Strategies for Electrocatalytic Water Splitting. Acc. Chem. Res..

[14] Panda A., Kim H. (2021). Phosphorus Embedded Mo-MXene/CQDs Hybrid: A 2D/0D Architecture for Bifunctional Electrochemical Water Splitting. Nanoscale.

[15] Panda A., Arumugasamy S.K., Lee J., Son Y., Yun K., Venkateswarlu S., Yoon M. (2021). Chemical-Free Sustainable Carbon Nano-Onion as a Dual-Mode Sensor Platform for Noxious Volatile Organic Compounds. Appl. Surf. Sci..

[16] Venkateswarlu S., Panda A., Kim E., Yoon M. (2018). Biopolymer-Coated Magnetite Nanoparticles and Metal-Organic Framework Ternary Composites for Cooperative Pb(II) Adsorption. ACS Appl. Nano Mater..

[17] Venkateswarlu S., Mahajan H., Panda A., Lee J., Govindaraju S., Yun K., Yoon M. (2021). Fe_3_O_4_ Nano Assembly Embedded in 2D-Crumpled Porous Carbon Sheets for High Energy Density Supercapacitor. Chem. Eng. J..

[18] Zhou H.C., Long J.R., Yaghi O.M. (2012). Introduction to Metal-Organic Frameworks. Chem. Rev..

[19] Furukawa H., Cordova K.E., O’Keeffe M., Yaghi O.M. (2013). The Chemistry and Applications of Metal-Organic Frameworks. Science.

[20] Tao Z., Wang T., Wang X., Zheng J., Li X. (2016). MOF-Derived Noble Metal Free Catalysts for Electrochemical Water Splitting. ACS Appl. Mater. Interfaces.

[21] Li F.L., Shao Q., Huang X., Lang J.P. (2018). Nanoscale Trimetallic Metal–Organic Frameworks Enable Efficient Oxygen Evolution Electrocatalysis. Angew. Chem.—Int. Ed..

[22] Zhao S., Wang Y., Dong J., He C.T., Yin H., An P., Zhao K., Zhang X., Gao C., Zhang L. (2016). Ultrathin Metal-Organic Framework Nanosheets for Electrocatalytic Oxygen Evolution. Nat. Energy.

[23] Chen L., Xu Q. (2019). Metal-Organic Framework Composites for Catalysis. Matter.

[24] Wang C., Liu S., Wang D., Chen Q. (2018). Interface Engineering of Ru-Co3O4 Nanocomposites for Enhancing CO Oxidation. J. Mater. Chem. A Mater..

[25] Gopi S., Panda A., Ramu A.G., Theerthagiri J., Kim H., Yun K. (2021). Bifunctional Electrocatalysts for Water Splitting from a Bimetallic (V Doped-NixFey) Metal–Organic Framework MOF@Graphene Oxide Composite. Int. J. Hydrogen Energy.

[26] Zhang B., Zheng Y., Ma T., Yang C., Peng Y., Zhou Z., Zhou M., Li S., Wang Y., Cheng C. (2021). Designing MOF Nanoarchitectures for Electrochemical Water Splitting. Adv. Mater..

[27] Khalid M., Hassan A., Honorato A.M.B., Crespilho F.N., Varela H. (2018). Nano-Flocks of a Bimetallic Organic Framework for Efficient Hydrogen Evolution Electrocatalysis. Chem. Commun..

[28] Zhou W., Wu Y.P., Wang X., Tian J.W., Huang D.D., Zhao J., Lan Y.Q., Li D.S. (2018). Improved Conductivity of a New Co(Ii)-MOF by Assembled Acetylene Black for Efficient Hydrogen Evolution Reaction. CrystEngComm.

[29] Rui K., Zhao G., Lao M., Cui P., Zheng X., Zheng X., Zhu J., Huang W., Dou S.X., Sun W. (2019). Direct Hybridization of Noble Metal Nanostructures on 2D Metal-Organic Framework Nanosheets to Catalyze Hydrogen Evolution. Nano Lett..

[30] Cao C., Ma D.D., Xu Q., Wu X.T., Zhu Q.L. (2019). Semisacrificial Template Growth of Self-Supporting MOF Nanocomposite Electrode for Efficient Electrocatalytic Water Oxidation. Adv. Funct. Mater..

[31] Chen B., Yang Z., Zhu Y., Xia Y. (2014). Zeolitic Imidazolate Framework Materials: Recent Progress in Synthesis and Applications. J. Mater. Chem. A Mater..

[32] Xie W., Gao C., Li J. (2021). Sustainable Biodiesel Production from Low-Quantity Oils Utilizing H6PV3MoW8O40 Supported on Magnetic Fe3O4/ZIF-8 Composites. Renew Energy.

[33] Mo Z., Tai D.Z., Zhang H., Shahab A. (2022). A Comprehensive Review on the Adsorption of Heavy Metals by Zeolite Imidazole Framework (ZIF-8) Based Nanocomposite in Water. Chem. Eng. J..

[34] Paul A., Banga I.K., Muthukumar S., Prasad S. (2022). Engineering the ZIF-8 Pore for Electrochemical Sensor Applications-A Mini Review. ACS Omega.

[35] Abdelhamid H.N. (2021). Zeolitic Imidazolate Frameworks (ZIF-8) for Biomedical Applications: A Review. Curr. Med. Chem..

[36] Park K.S., Ni Z., Côté A.P., Choi J.Y., Huang R., Uribe-Romo F.J., Chae H.K., O’Keeffe M., Yaghi O.M. (2006). Exceptional Chemical and Thermal Stability of Zeolitic Imidazolate Frameworks. Proc. Natl. Acad. Sci. USA.

[37] Chameh B., Moradi M., Hajati S., Hessari F.A. (2021). Design and Construction of ZIF(8 and 67) Supported Fe_3_O_4_ Composite as Advanced Materials of High Performance Supercapacitor. Phys. E Low Dimens. Syst. Nanostruct..

[38] Meng W., Chen W., Zhao L., Huang Y., Zhu M., Huang Y., Fu Y., Geng F., Yu J., Chen X. (2014). Porous Fe3O4/Carbon Composite Electrode Material Prepared from Metal-Organic Framework Template and Effect of Temperature on Its Capacitance. Nano Energy.

[39] Zhang Y.F., Qiu L.G., Yuan Y.P., Zhu Y.J., Jiang X., Xiao J.D. (2014). Magnetic Fe_3_O_4_@C/Cu and Fe3O4@CuO Core-Shell Composites Constructed from MOF-Based Materials and Their Photocatalytic Properties under Visible Light. Appl. Catal. B.

[40] Zhou Q., Xing J., Gao Y., Lv X., He Y., Guo Z., Li Y. (2014). Ordered Assembly of NiCo_2_O_4_ Multiple Hierarchical Structures for High-Performance Pseudocapacitors. ACS Appl. Mater. Interfaces.

[41] Cai D., Wang D., Liu B., Wang Y., Liu Y., Wang L., Li H., Huang H., Li Q., Wang T. (2013). Comparison of the Electrochemical Performance of Nimoo4 Nanorods and Hierarchical Nanospheres for Supercapacitor Applications. ACS Appl. Mater. Interfaces.

[42] Xiang C., Li M., Zhi M., Manivannan A., Wu N. (2013). A Reduced Graphene Oxide/Co_3_O_4_ Composite for Supercapacitor Electrode. J. Power Sources.

[43] Yu W., Jiang X., Ding S., Li B.Q. (2014). Preparation and Electrochemical Characteristics of Porous Hollow Spheres of NiO Nanosheets as Electrodes of Supercapacitors. J. Power Sources.

[44] Yang J., Lian L., Ruan H., Xie F., Wei M. (2014). Nanostructured Porous MnO_2_ on Ni Foam Substrate with a High Mass Loading via a CV Electrodeposition Route for Supercapacitor Application. Electrochim. Acta.

[45] Tan J.B., Sahoo P., Wang J.W., Hu Y.W., Zhang Z.M., Lu T.B. (2018). Highly Efficient Oxygen Evolution Electrocatalysts Prepared by Using Reduction-Engraved Ferrites on Graphene Oxide. Inorg. Chem. Front..

[46] Li M., Xiong Y., Liu X., Bo X., Zhang Y., Han C., Guo L. (2015). Facile Synthesis of Electrospun MFe_2_O_4_ (M = Co, Ni, Cu, Mn) Spinel Nanofibers with Excellent Electrocatalytic Properties for Oxygen Evolution and Hydrogen Peroxide Reduction. Nanoscale.

[47] Wang Z., Liu X., Lv M., Chai P., Liu Y., Meng J. (2008). Preparation of Ferrite MFe2O4 (M = Co, Ni) Ribbons with Nanoporous Structure and Their Magnetic Properties. J. Phys. Chem. B.

[48] Cui L., Qu F., Liu J., Du G., Asiri A.M., Sun X. (2017). Interconnected Network of Core–Shell CoP@CoBiPi for Efficient Water Oxidation Electrocatalysis under Near Neutral Conditions. ChemSusChem.

[49] Wang H.Y., Hung S.F., Chen H.Y., Chan T.S., Chen H.M., Liu B. (2016). In Operando Identification of Geometrical-Site-Dependent Water Oxidation Activity of Spinel Co_3_O_4_. J. Am. Chem. Soc..

[50] Zhu Y.P., Ma T.Y., Jaroniec M., Qiao S.Z. (2017). Self-Templating Synthesis of Hollow Co_3_O_4_ Microtube Arrays for Highly Efficient Water Electrolysis. Angew. Chem.—Int. Ed..

[51] Hu H., Guan B., Xia B., Lou X.W. (2015). Designed Formation of Co_3_O_4_/NiCo_2_O_4_ Double-Shelled Nanocages with Enhanced Pseudocapacitive and Electrocatalytic Properties. J. Am. Chem. Soc..

[52] Liu Y., Li J., Li F., Li W., Yang H., Zhang X., Liu Y., Ma J. (2016). A Facile Preparation of CoFe2O4 Nanoparticles on Polyaniline-Functionalised Carbon Nanotubes as Enhanced Catalysts for the Oxygen Evolution Reaction. J. Mater. Chem. A Mater..

[53] Kargar A., Yavuz S., Kim T.K., Liu C.H., Kuru C., Rustomji C.S., Jin S., Bandaru P.R. (2015). Solution-Processed CoFe2O4 Nanoparticles on 3D Carbon Fiber Papers for Durable Oxygen Evolution Reaction. ACS Appl. Mater. Interfaces.

[54] Lu X.F., Gu L.F., Wang J.W., Wu J.X., Liao P.Q., Li G.R. (2017). Bimetal-Organic Framework Derived CoFe_2_O_4_/C Porous Hybrid Nanorod Arrays as High-Performance Electrocatalysts for Oxygen Evolution Reaction. Adv. Mater..

[55] Shanmugavani A., Kalpana D., Selvan R.K. Electrochemical Properties of CoFe2O4 Nanoparticles as Negative and Co(OH)_2_ and Co_2_Fe(CN)_6_ as Positive Electrodes for Supercapacitors. https://reader.elsevier.com/reader/sd/pii/S0025540815002664?token=F9FC8EBC3DB2D9B8F6097010A2361E59DAEE1F164F815537E592D646AE6C02130E77D64EECC7AAB7A8CDE5809A2B6EE5&originRegion=us-east-1&originCreation=20210831033308.

[56] Ortiz-Quiñonez J.L., Pal U. (2019). Borohydride-Assisted Surface Activation of Co_3_O_4_/CoFe_2_O_4_ Composite and Its Catalytic Activity for 4-Nitrophenol Reduction. ACS Omega.

[57] Kubisztal J., Kubisztal M. (2022). Synthesis and Characterisation of Cobalt Ferrite Coatings for Oxygen Evolution Reaction. Catalysts.

[58] Ferreira L.S., Silva T.R., Santos J.R., Silva V.D., Raimundo R.A., Morales M.A., Macedo D.A. (2019). Structure, magnetic behavior and OER activity of CoFe_2_O_4_ powders obtained using agar-agar from red seaweed (Rhodophyta). Mater. Chem. Phys..

[59] Silva V.D., Ferreira L.S., Simões T.A., Medeiros E.S., Macedo D.A. (2019). 1D hollow MFe_2_O_4_ (M = Cu, Co, Ni) fibers by Solution Blow Spinning for oxygen evolution reaction. J. Colloid Interface Sci..

[60] Sagu J.S., Mehta D., Wijayantha K.U. (2018). Electrocatalytic activity of CoFe_2_O_4_ thin films prepared by AACVD towards the oxygen evolution reaction in alkaline media. Electrochem. Commun..

[61] Mahala C., Sharma M.D., Basu M. (2018). 2D nanostructures of CoFe_2_O_4_ and NiFe_2_O_4_: Efficient oxygen evolution catalyst. Electrochim. Acta.

[62] Zhang Z., Zhang J., Wang T., Li Z., Yang G., Bian H., Li J., Gao D. (2018). Durable oxygen evolution reaction of one dimensional spinel CoFe_2_O_4_ nanofibers fabricated by electrospinning. RSC Adv..

[63] Liu T., Li P., Yao N., Kong T., Cheng G., Chen S., Luo W. (2019). Self-sacrificial template-directed vapor-phase growth of MOF assemblies and surface vulcanization for efficient water splitting. Adv. Mater..

[64] Wang J., Zhang M., Li J., Jiao F., Lin Y., Gong Y. (2020). A highly efficient electrochemical oxygen evolution reaction catalyst constructed from a S-treated two-dimensional Prussian blue analogue. Dalton Trans..

[65] He P., Xie Y., Dou Y., Zhou J., Zhou A., Wei X., Li J.R. (2019). Partial sulfurization of a 2D MOF array for highly efficient oxygen evolution reaction. ACS Appl. Mater. Interfaces.

[66] Zou Z., Wang T., Zhao X., Jiang W.J., Pan H., Gao D., Xu C. (2019). Expediting in-situ electrochemical activation of two-dimensional metal–organic frameworks for enhanced OER intrinsic activity by iron incorporation. ACS Catal..

[67] Gao Z., Yu Z.W., Liu F.Q., Yang C., Yuan Y.H., Yu Y., Luo F. (2019). Stable Iron Hydroxide Nanosheets@ Cobalt-Metal–Organic–Framework Heterostructure for Efficient Electrocatalytic Oxygen Evolution. ChemSusChem.

[68] Liu K., Zhang C., Sun Y., Zhang G., Shen X., Zou F., Zhang H., Wu Z., Wegener E.C., Taubert C.J. (2018). High-performance transition metal phosphide alloy catalyst for oxygen evolution reaction. ACS Nano.

