# Supplementary material for: A Green Synthesis of CoFe2O4 Decorated ZIF-8 Composite for Electrochemical Oxygen Evolution
Source: Int J Mol Sci. 2023 May 31;24(11):9585. doi: 10.3390/ijms24119585 (PMC10253966; doi:10.3390/ijms24119585)
Supplement: Supplementary file 1 [file ijms-24-09585-s001.zip › ijms-2417003-supplementary.pdf]

Supplementary Materials

A Green Synthesis of $CoFe_2O_4$ Decorated ZIF-8 Composite for Electrochemical Oxygen Evolution

Atanu Panda, Hang-Kyu Cho and Hansang Kim *

Department of Mechanical Engineering, Gachon University, Seongnam-si 13120, Gyeonggi-do, Republic of Korea; santu.atanu.panda@gmail.com (A.P.); 5318@gachon.ac.kr (H.-K.C.)
* Correspondence: hskim70@gachon.ac.kr (H.K)

Keywords: spinel ferrite; ZIF-8; magnetic; $CoFe_2O_4$; oxygen evolution reaction

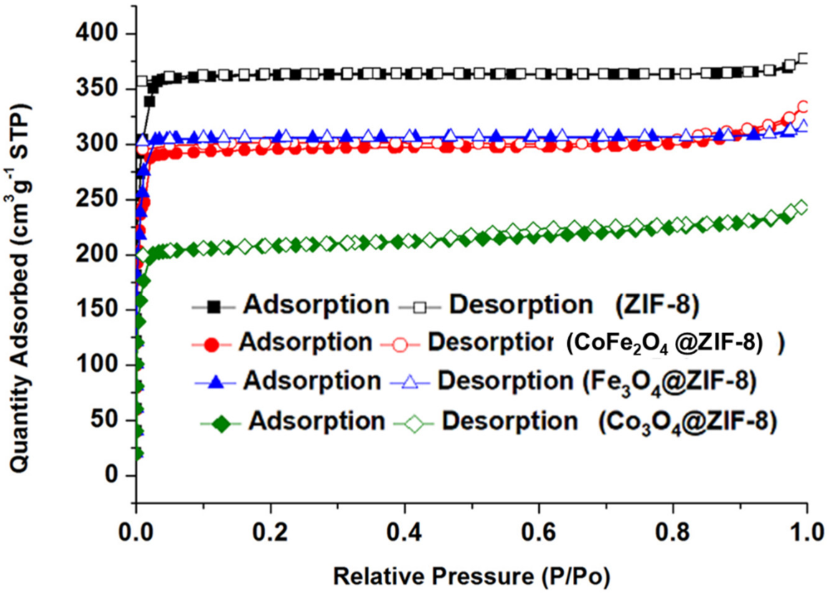

Figure S1. BET surface area analysis in N_2.

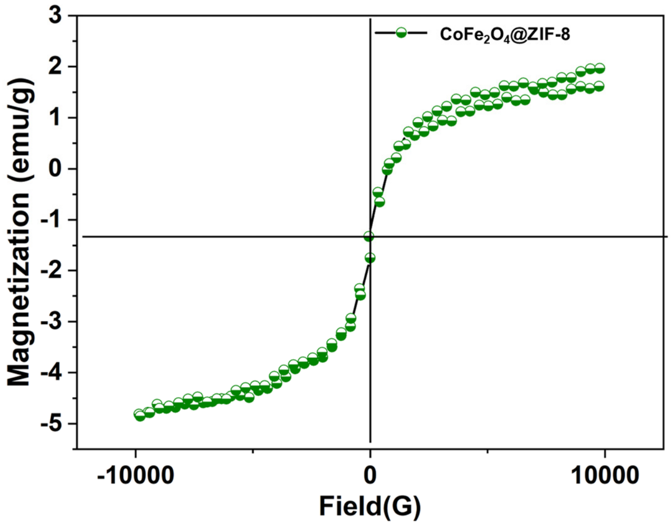

Figure S2. VSM of $CoFe_2O_4$@ZIF-8

Elem	Weight %	Atomic %
C K	48.6	83.2
FeK	8.9	3.3
CoK	3	1
ZnK	39.6	12.5
Total	100	100

Figure S3. EDS elemental composition of $CoFe_2O_4$@ZIF-8.

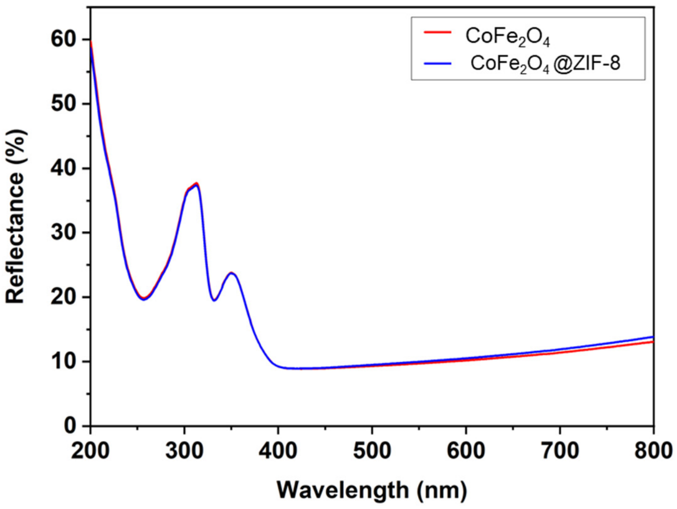

Figure S4. UV-vis-DRS analysis of $CoFe_2O_4$ and $CoFe_2O_4$@ZIF-8

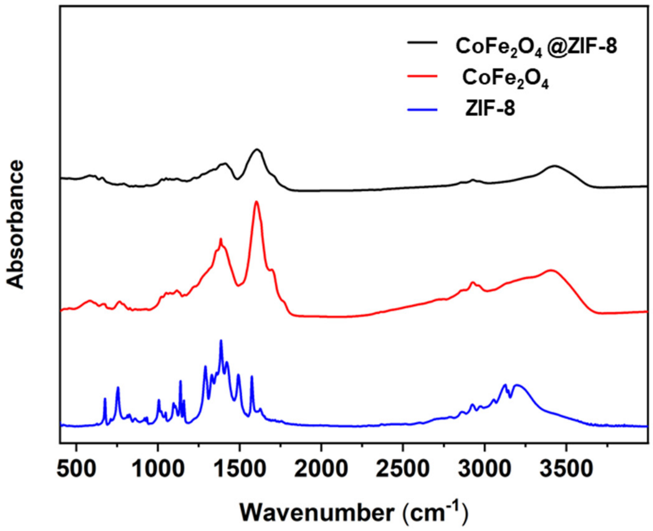

Figure S5. FT-IR analysis of ZIF-8, $CoFe_2O_4$ and $CoFe_2O_4$@ZIF-8 samples

Table S1. EDS analysis of ZIF-8, $CoFe_2O_4$ and $CoFe_2O_4$@ZIF-8 samples

Sample	Chemical	ms%
ZIF-8	C	68.67
	O	28.72
	Zn	2.62
$CoFe_2O_4$	C	65.90
	O	28.88
	Fe	2.48
	Co	0.41

Table S2. A list of average pore diameter from BET measurements.

	Average pore diameter (Å)
ZIF-8	11.7
$CoFe_2O_4$	24
$CoFe_2O_4$@ZIF-8	32

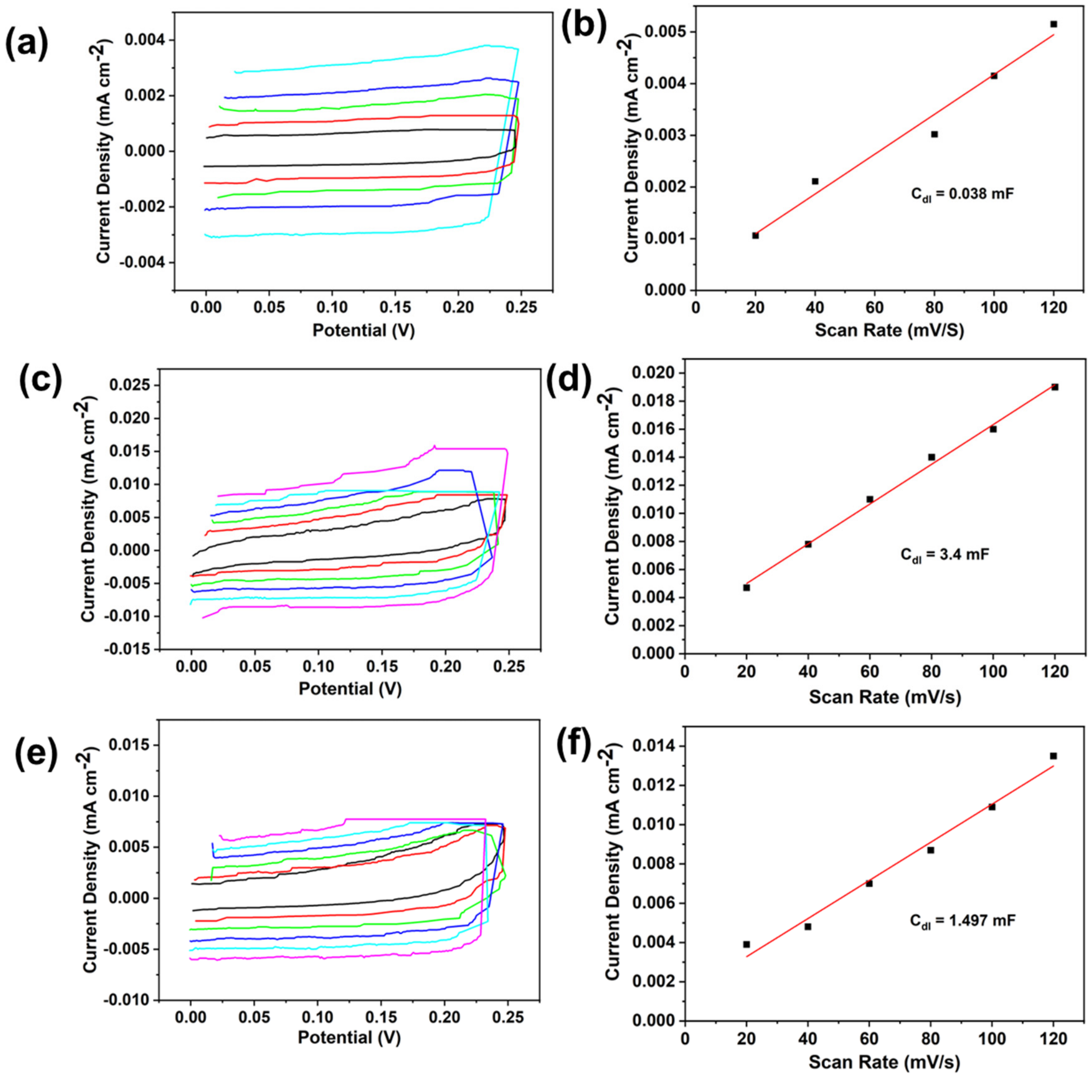

Figure S6. Double layer capacitance and respective CVs of (a,b) bare CPE electrode, (c,d) ZIF-8, (e,f) $CoFe_2O_4$ at scan rate 20-120 mV s^{-1}.

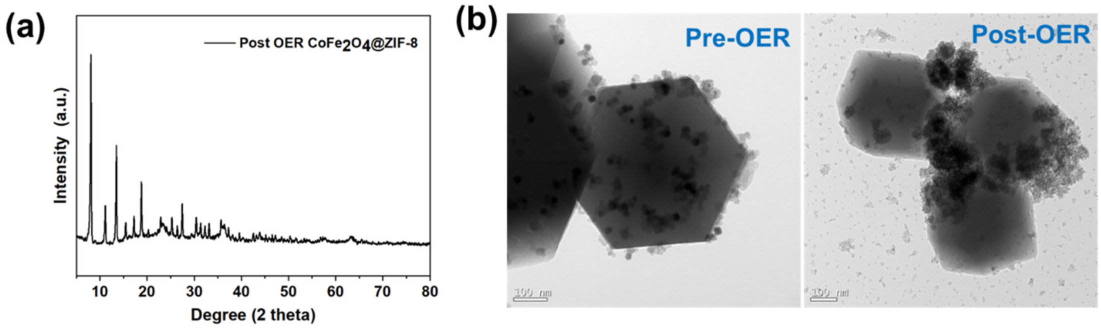

Figure S7. Pre and Post OER characterization of $CoFe_2O_4$@ZIF-8 composite; (a) PXRD, (b) TEM images.
